# Viral loads, lymphocyte subsets and cytokines in asymptomatic, mildly and critical symptomatic patients with SARS-CoV-2 infection: a retrospective study

**DOI:** 10.1186/s12985-021-01597-x

**Published:** 2021-06-12

**Authors:** Shi-Wei Yin, Zheng Zhou, Jun-Ling Wang, Yun-Feng Deng, Hui Jing, Yi Qiu

**Affiliations:** 1 Shandong Provincial Public Health Clinical Center, Katharine Hsu International Research Institute of Infectious Disease, Shandong University Affiliated Hospital, Jinan, 250013 Shandong People’s Republic of China; 2 Department of Clinical Laboratory, Shandong Provincial Public Health Clinical Center, Shandong University Affiliated Hospital, Jinan, 250013 Shandong People’s Republic of China; 3 Key Laboratory of Birth Regulation and Control Technology of National Health Commission of China, Maternal and Child Health Care Hospital of Shandong Province, 238 East Road of Jingshi, Jinan, 250014 Shandong People’s Republic of China

**Keywords:** Asymptomatic, COVID-19, Interleukin, Lymphocyte subset, SARS-CoV-2, Viral load

## Abstract

**Background:**

Tens of million cases of coronavirus disease-2019 (COVID-19) have occurred globally. The severe acute respiratory syndrome coronavirus 2 (SARS-CoV-2) attacks the respiratory system, causing pneumonia and lymphopenia in infected individuals. The aim of the present study is to investigate the laboratory characteristics of the viral load, lymphocyte subset and cytokines in asymptomatic individuals with SARS-CoV-2 infection in comparison with those in symptomatic patients with COVID-19.

**Methods:**

From January 24, 2020, to April 11, 2020, 48 consecutive subjects were enrolled in this study. Viral loads were detected by RT-PCR from throat-swab, sputum and feces samples. Lymphocyte subset levels of CD3 + , CD4 + , and CD8 + T lymphocytes, B cells and NK cells were determined with biological microscope and flow cytometric analysis. Plasma cytokines (IL2, IL4, IL5, IL6, IL8, IL10, TNF-α, IFN-α and IFN-γ) were detected using flow cytometer. Analysis of variance (ANOVA), Chi-square or Fisher's exact test and Pearson’s Correlation assay was used for all data.

**Results:**

Asymptomatic (AS), mild symptoms (MS) and severe or critical cases (SCS) with COVID-19 were 11 (11/48, 22.9%), 26 (54.2%, 26/48) and 11 cases (11/48, 22.9%), respectively. The mean age of AS group (47.3 years) was lower than SCS group (63.5 years) (*P* < 0.05). Diabetes mellitus in AS, MS and SCS patients with COVID-19 were 0, 6 and 5 cases, respectively, and there was a significant difference between AS and SCS (*P* < 0.05). No statistical differences were found in the viral loads of SARS-CoV-2 between AS, MS and SCS groups on admission to hospital and during hospitalization. The concentration of CD 3 + T cells (*P* < 0.05), CD3 + CD4 + T cells (*P* < 0.05), CD3 + CD8 + T cells (*P* < 0.01), and B cells (*P* < 0.05) in SCS patients was lower than in AS and MS patients, while the level of IL-5 (*P* < 0.05), IL-6 (*P* < 0.05), IL-8 (*P* < 0.01) and IL-10 (*P* < 0.01), and TNF-α (*P* < 0.05) was higher. The age was negatively correlated with CD3 + T cells (*P* < 0.05), CD3 + CD4 + T cells (*P* < 0.05), and positively correlated with IL-2 (*P* < 0.001), IL-5 (*P* < 0.05), IL-6 (*P* < 0.05) IL-8 (*P* < 0.05), and IL-10 (*P* < 0.05). The viral loads were positively correlated with IL-2 (*P* < 0.001), IL-5 (*P* < 0.05), IL-6 (*P* < 0.05) IL-8 (*P* < 0.05) and IL-10 (*P* < 0.05), while negatively correlated with CD 3 + T cells (*P* < 0.05) and CD3 + CD4 + T cells (*P* < 0.05).

**Conclusions:**

The viral loads are similar between asymptomatic, mild and severe or critical patients with COVID-19. The severity of COVID-19 may be related to underlying diseases such as diabetes mellitus. Lymphocyte subset and plasma cytokine levels may be as the markers to distinguish severely degrees of disease, and asymptomatic patients may be as an important source of infection for the COVID-19.

## Introduction

Severe acute respiratory syndrome coronavirus 2 (SARS-CoV-2) is a new family of coronaviruses that can cause public health emergency of the new infectious primary atypical pneumonia pandemic [1, 2]. The disease caused by SARS-CoV-2 which named Coronavirus Disease-2019 (COVID-19) by the World Health Organization (WHO) [2–4]. Laboratory diagnosis methods for COVID-19 mainly include pathogenic examination based on viral nucleic acid, serological examination through antibody detection, and general examination [5, 6]. The abnormal immune system and cytokine storm have been detected, such as the numbers of total T-cells, CD4 + T-cells and CD8 + T-cells are decreased, and the surviving T-cells are functionally exhausted [4], while the leukocytes, neutrophils, infection biomarkers and the concentrations of cytokines [interleukin (IL)-2R, IL-6, IL-8, IL-10 and tumor necrosis factor (TNF)-α] were significantly increased in patients with SARS-CoV-2 infection [5, 6]. A consumption of CD4 + and CD8 + T cells might explain the aggravated inflammatory response, the aforementioned cytokine storm activation and worse infection prognosis [7, 8]. Cytokine storms are one of the most important mechanisms underlying disease progression and death, and overstimulate the body’s immune response of inflammatory factors. Besides mild, severe and critical cases, many individuals are likely asymptomatic but potentially carry the SARS-CoV-2 [9]. The viral transmission have considered the potential contribution of the asymptomatic carriers. Asymptomatic cases have not symptoms of fever, dry cough, fatigue and abnormal chest computed tomography (CT) findings [7]. Therefore, for early detection, early isolation, and early treatment to prevent the spread of COVID-19, the detection and diagnosis of asymptomatic SARS-CoV-2 carriers are very important.

The aim of the present study was to investigate the laboratory characteristics of the viral load, CD3 + T-cells, CD4 + T-cells and CD8 + T-cells and cytokines in asymptomatic individuals with SARS-CoV-2 infection in comparison with those in symptomatic patients with COVID-19.

## Methods

### Patients

This study was conducted at the infectious disease department, Shandong Provincial Chest Hospital, Jinan, China, from January 24, 2020, to April 11, 2020, and the final date of follow-up was April 25, 2020. We retrospectively reviewed documents on epidemiological investigation and medical records. Asymptomatic carriers, mild and critical cases were screened from close contacts in laboratory confirmed COVID-19, diagnosed using reverse transcriptase–polymerase chain reaction (RT-PCR) (ABI 7500, Thermo Fisher, Massachusetts, USA) and clinical symptoms. The day of first positive PCR test was from the date of blood sampling. The day of symptom onset such as fever (> 37.8 °C), chills, myalgia, fatigue, nasal congestion, hypogeusia, sore throat, dyspnoea, cough, sputum production, headache and igestive symptoms was from 1 to 5 days. Asymptomatic individuals were defined as those who did not report any of the following symptoms: fever (> 37.8 °C), chills, myalgia, fatigue, rhinorrhea, nasal congestion, hyposmia, hypogeusia, sore throat, dyspnoea, cough, sputum production, haemoptysis, headache, dizziness, anorexia, nausea, vomiting, abdominal pain and diarrhoea. The Guidance for Corona Virus Disease 2019: Prevention, Control, Diagnosis and Management (sixth edition), which was issued by China’s National Health Commission, was used to classify the patients with mild, and critical disease according to the severity of the COVID-19 symptoms. Patients with (1) mild disease present with mild symptoms only without radiographic features; (2) severe or critical disease meet one of the following three criteria, namely, dyspnea, which is defined as a respiratory rate > 30 times/min, an oxygen saturation of < 93% in ambient air, or a ratio of arterial oxygen partial pressure to fractional inspired oxygen < 300 mmHg; and respiratory failure or septic shock, or multiple organ failure [10].

From 24 January 2020 to 11 April 2020, 48 consecutive subjects, comprising 26 males and 22 female patients, who were aged from 18 to 86 years and were from Shandong Provincial Chest hospital (Provincial Public Health Clinical Center), China, were enrolled in this study. The patients’ epidemiological data, demographic data, clinical characteristics, radiographic characteristics, and key laboratory parameters were analyzed. This study was approved by the ethics committee of Shandong Provincial Chest Hospital (Provincial Public Health Clinical Center), Shandong, China (2020XKYYEC-03, February 10, 2020), and it conformed to the ethical principles of the Declaration of Helsinki. According to the Guidance for Corona Virus Disease 2019: Prevention, Control, Diagnosis and Management [10], alpha-interferon, ribavirin, lopinavir/ritonavir, chloroquine phosphate, and arbidol were to be tried for all 48 patients with COVID-19.

### Sample acquisition

All sample acquisitions for follow-up RT-PCR at the care centre were carried out by experienced physicians donning the complete personal protective equipment. The sample acquisition time was 1 day before admission (diagnosis date) and 7 days of hospitalization. Throat-swab specimens were collected from all patients and the samples were maintained in a viral-transport medium for laboratory testing. Specimens, including sputum or bronchoalveolar lavage fluid, blood, urine, and feces, were cultured to identify pathogenic bacteria or fungi that may be associated with the SARS-CoV-2 infection. Sputum specimens were also collected in some patients. The nasopharyngeal samples were obtained in which swabs were inserted through the nostril to a distance equivalent to the outer opening of the ear canal and gently rubbed for several seconds to absorb the secretions. For oropharyngeal samples, the tonsillar pillars were swabbed. The nasopharynx and oropharynx were swabbed once each with separate swabs, placed in a single tube containing 300 µL of viral transport medium and transported to our laboratory under cold conditions for subsequent RNA extraction and RT-PCR testing.

Individuals with asymptomatic COVID-19 were laboratory-confirmed as positive for SARS-CoV-2 by testing the nasopharynx and oropharynx samples for SARS-CoV-2 nucleic acids; these individuals did not show any obvious symptoms during nucleic acid screening. The asymptomatic individuals were identified mainly by investigating clusters of outbreaks and tracking infectious individuals whose computed tomography (CT) images were normal and who had no symptoms on admission to hospital or during hospitalization. We combined the patients with severe or critical disease into one group for further analysis, because of the small numbers of patients present in each group. Therefore, our study comprised three groups, namely, 11 asymptomatic carriers, 26 mild cases, and 11 severe or critical cases of COVID-19.

### RT-PCR for SARS-CoV-2 genes

All specimens were handled in a biosafety cabinet, and laboratory confirmation of the SARS-CoV-2 was performed by the Local Centers for Disease Control and Prevention (CDC) according to Chinese CDC protocol. Viral RNA from the upper respiratory tract swab samples was extracted and purified from Tian Long technology nucleic acid extraction (NP968-C, Tian Long, Xian). SARS-CoV-2 was used nucleic acid detection kit (fluorescent PCR method), this kit is based on one-step RT-PCR technology, selected SARS-CoV-2 ORF1Ab and N genes as amplification target region, specific primers and fluorescent probes for the detection of novel Coronavirus RNA in samples. Samples were collected in accordance with the “Guidelines for Laboratory Detection of Coronavirus Infected Pneumonia” published by the National Health and Development Commission, and PCR amplification was performed using ABI7500 instrument which was approved by the China Food and Drug Administration, according to the manufacturer’s instructions. In brief, RNA was extracted from clinical samples with commercial RNA extraction kits. Then, 5 μl of RNA template was used for real‐time RT–PCR, which targeted the open reading frame (ORF)1ab and nucleoprotein gene. Conditions for applications of real‐time PCR (RT–PCR) were as follows: 50 °C for 15 min and 95 °C for 5 min, 45 cycles of amplification at 95 °C for 10 s and 55 °C for 45 s. The positive COVID‐19 real‐time RT–PCR result was defined if both ORF1ab and nucleoprotein cycle thresholds were < 40.

### The laboratory analysis of CD3 + , CD4 + , CD8 + B cell and NK cell, T lymphocytes and plasma cytokines (IL2, IL4, IL6, IL8, IL10, TNF—α and IFN—γ)

Lymphocyte subset levels of (CD)3 + , CD4 + , and CD8 + T lymphocytes, B cells and NK cells were determined with biological microscope and flow cytometry. BD Multi-test TM IMK KIT (Becton, Dickinson and Company, BD Biosciences, USA) was used for lymphocyte subset analysis, and lymphocyte subset levels, that was, cluster of differentiation CD3 + , CD4 + , and CD8 + T lymphocytes. In brief, 20-µL blood specimens were diluted with 380 µL of assay diluent solution. Five microliters of each diluted blood specimen was drawn onto the coated working surface of the chips and incubated for 40 min in a humidified enclosure. The chips were inserted into the staining holder, and the blood was washed from the chips using diluent solution. The chips were stained with staining solution for 1 min, and were then transferred into hydrogen peroxide working solution and submerged for 4 min. The chips were washed with 75% alcohol solution, dried under an air outlet for 6 min, stained with counterstaining solution for 1 min, washed with water after submerging for 30 s and dried again under an air outlet. Lymphocytes were automatically scanned and counted with an accessorized biological microscope (BM2000, Nanjing Jiangnan Novel Optics Co., Ltd, China. UI-1240, IDS, Germany; MS-300, Nanjing Red, Green and Blue Intelligent Systems Co., Ltd., China) and software.

Plasma cytokines (IL-2, IL-4, IL-5, IL-6, IL-8, IL-10, IL-12p70, IL-17, TNF-α, IFN-α and IFN-γ) were detected with Twelve Cytokines Assay Kit (multi-microsphere flow immunofluorescence, Qingdao Raisecare Biological Technology Co., Ltd.). Samples were analyzed on the BD FACSAria™ II system with BD FACSDiva software and LENGENDplexTM software (v8.0). All tests were performed according to the product manual. All plasma samples were freshly isolated and detected according to the manufacturer’s manual. Briefly, first, 25 µl of experimental buffer, 25 µl of centrifuged plasma, 25 µl of capture Ab, and 25 µl of detection Ab were added to detection tubes consecutively. Next, all tubes were incubated at room temperature in a shaking incubator for 2 h (400–500 rpm). After that, 25 µl SA‐PE (streptavidin labeled with phycoerythrin) was added to each tube and incubated at the same condition as above for 30 min. Then, 500 µl diluted wash buffer was added to each tube. After a brief vortexing, the detection tubes were centrifuged at 300 × *g* for 5 min. Supernatant was discarded, and the clustered microspheres were suspended with 200 µl diluted washing buffer. Florescence intensity was measured by a flow cytometer (Canto II, BD, USA) and the cytokine concentrations were calculated according to manufacturer’s instruction.

### Statistical analysis

All statistical analyses were carried out in IBM SPSS Statistics for Windows, 22.0. The results are presented as mean ± standard deviation (s.d.). Descriptive analysis focused on frequencies, and percentages while chi-square tests, for independent samples, *t*-tests and one-way analysis of variance (ANOVA) were utilised to determine the differences between groups for selected demographic variables. We compared variables across groups using Student’s *t* test and ANOVA for numerical variables, and Chi-squared test or Fisher exact test for categorical variables. The data such as ages, viral loads, levels of inflammatory markers and lymphocyte subsets, was normally distributed. To test the difference between the means of several subgroups of a variable (multiple testing), One-way ANOVA was used. Classification variables are expressed in frequency or percentage, and significance was detected by chi square or Fisher's exact test. Pearson’s Correlation assay was used for correlation between the age, viral loads, and levels of inflammatory markers and lymphocyte subsets. Statistical significance was determined as *P* < 0·05.

## Results

### The baseline and clinical characteristics of 48 subjects

In the present study, 11 individuals remained asymptomatic (AS) (no systemic, upper respiratory, gastrointestinal and lung symptoms, and chest CT changes) (11/48, 22.9%; 7 males, 7/11, 63.6%), 26 patients with mild symptoms (MS) (26/48, 54.2%, and 13 males, 13/26, 50.0%) (mild systemic, upper respiratory, gastrointestinal and lung symptoms and without radiographic features), and 11 cases with severe or critical cases (SCS) (11/48, 22.9%; 5 males, 5/11, 45.5%) with COVID-19 were investigated. None of the AS who were infected with SARS-CoV-2 had symptoms on admission to hospital or during hospitalization. The clinical characteristics of 26 MS and 11 SCS with SARS-CoV-2 are presented in Table 1. Systemic symptoms include: fever, chills, lymph-node enlargement, rash and headache, fatigue, myalgia or arthralgia. Upper respiratory symptoms include: nasal congestion, sore throat, throat congestion and swollen tonsils. Gastrointestinal symptoms include: nausea or vomiting and diarrhea. Lung symptomsinclude: cough, sputum production, hemoptysis and shortness of breath. Chest CT was computed tomography changes. Statistical differences were found between the AS group and MS and SCS groups regarding the systemic symptoms (*P* = 0.002), upper respiratory symptoms (*P* = 0.001) and lung symptoms and CT changes (*P* < 0.001). The ages of SCS (63.5 ± 13.1) were significantly higher than AS [(45.0 ± 14.5) and (47.3 ± 17.6) (F = 4.789, *P* = 0.013)].

**Table 1:** Clinical characteristics of the study subjects according to the disease severity of COVID-19

Groups	Systemic	Upper respiratory	Gastrointestinal	Lung	CT
Mild cases (n = 26)	14 (53.8%)	20 (76.9%)	2 (7.7%)	16 (61.5%)	0 (0%)
Severe or critical cases (n = 11)	11 (100%)	11 (100%)	4 (36.4%)	11 (100%)	11(100%)
Chi-Square value	7.514	3.030	4.667	5.798	16.865
*P* value	0.007	0.151	0.051	0.018	0.000

Categorical variables were compared using the Fisher’s exact test

Lung = Lung symptoms include: cough, sputum production, hemoptysis and shortness of breath

CT, changes of chest computed tomography

The prevalence of diabetes mellitus, hypertension and tuberculosis in AS, MS and SCS are presented in Table 2. Diabetes mellitus was 0, 6 and 5 cases in AS group, MS group and SCS group, respectively. Statistical differences were found between the AS group and MS and SCS groups (n = 37) regarding diabetes mellitus (*P* = 0.048) (see Table 2), and between AS group and SCS group (*x*^2^ = 6.471, *P* = 0.035). Hypertension was 2, 8 and 4 cases, and tuberculosis was 0, 1 and 1 case in the AS group, MS group and SCS group, respectively. There was no difference in the prevalence of hypertension and tuberculosis between AS group, MS group and SCS groups (*P* > 0.05).

**Table 2:** Diabetes mellitus, hypertension and tuberculosis in asymptomatic, mild symptoms and severe or critical cases of COVID-19

Groups	Diabetes mellitus	Hypertension	Tuberculosis
Asymptomatic cases (n = 11)	0 (0%)	2 (18.2%)	0 (0%)
Mild and severe cases (n = 37)	11 (29.7%)	12 (32.4%)	2 (5.4%)
Chi-Square value	4.243	0.833	0.860
*P* value	0.048	0.469	0.499

Categorical variables were compared using the Fisher’s exact test

### The viral load, levels of inflammatory markers and lymphocyte subsets in 48 subjects on admission to hospital

Table 3 presents the results from the viral load, inflammatory markers and lymphocyte subset assays on throat swabs, sputum and feces from the subjects infected with SARS-CoV-2 on admission to hospital. The mean Ct values of the SARS-CoV-2 genes in AS, MS and SCS were not significant difference. Statistical differences were evident among the three groups regarding the CD 3 + T cells (*P* < 0.05), B cells (*P* < 0.01) and inflammatory cytokines levels, including IL-5 (*P* < 0.05), IL-6 (*P* < 0.05), IL-8 (*P* < 0.01) and IL-10 (*P* < 0.01), and TNF-α (*P* < 0.05).

**Table 3:** The viral load, levels of inflammatory markers and lymphocyte subsets of the study subjects according to disease severity of COVID-19 on admission to hospital

Laboratory findings	Disease severity of COVID-19			Statistic parameter	
(mean ± SD)	AS (n = 11)	MS (n = 26)	SCS (n = 11)	*F* value	*P* value
Ct values	31.9 ± 2.6	31.0 ± 3.0	31.6 ± 2.8	0.398	0.674
B cells (%)	28.9 ± 14.8	13.3 ± 7.7	9.6 ± 6.6	7.018	0.002**
NK cells (%)	6.6 ± 5.1	3.8 ± 1.8	3.5 ± 1.1	1.286	0.286
CD 3 + T cells (%)	61.7 ± 7.8	63.9 ± 12.1	52.1 ± 13.2	4.079	0.024*
CD3 + CD4 + T cells (%)	33.3 ± 5.6	36.1 ± 9.7	30.3 ± 8.7	1.764	0.183
CD3 + CD8 + T cells (%)	23.1 ± 6.3	24.2 ± 10.5	17.0 ± 5.6	2.634	0.083
CD4 + /CD8 + ratio (%)	1.6 ± 0.8	1.8 ± 0.9	1.9 ± 0.8	1.764	0.183
IL2 (pg/mL)	4.8 ± 6.2	4.8 ± 4.1	8.3 ± 6.7	1.834	0.172
IL4 (pg/mL)	3.8 ± 2.6	3.8 ± 1.8	3.4 ± 1.1	0.161	0.852
IL5 (pg/mL)	5.9 ± 6.4	5.3 ± 4.4	6.3 ± 4.9	0.179	0.836
IL6 (pg/mL)	7.0 ± 10.2	6.0 ± 11.3	572.7 ± 1326.4	3.479	0.039*
IL8 (pg/mL)	52.9 ± 123.0	88.8 ± 90.8	885.7 ± 1920.0	3.348	0.044*
IL10 (pg/mL)	2.9 ± 1.1	2.7 ± 0.8	7.0 ± 7.6	5.819	0006**
TNF-α (pg/mL)	19.3 ± 43.6	58.2 ± 60.8	9.5 ± 7.5	4.689	0.014*
INF-α (pg/mL)	4.0 ± 3.9	5.7 ± 9.3	3.4 ± 0.7	0.451	0.640
INF-γ (pg/mL)	16.1 ± 34.8	26.9 ± 66.9	11.5 + 8.3	0.390	0.680

One‐way analysis of variance. Statistically significant, *P < 0. 05, **P < 0.001

### The viral load, levels of inflammatory markers and lymphocyte subsets in 48 subjects during hospitalization

Table 4 presents the results from the viral load, inflammatory markers and lymphocyte subset assays from the subjects infected with SARS-CoV-2 during hospitalization 5 to 7 days. No significant difference in probability of the viral loads across the AS, MS and SCS groups were found. In addition, the levels of IL-6 and IL-10 in SCS COVID-19 patients were significantly higher than that in AS and MS group. Statistical differences were evident among the three groups regarding the B cells (*P* < 0.01), CD3 + T cells (*P* < 0.01), CD3 + CD4 + T cells (*P* < 0.05), CD3 + CD8 + T cells (*P* < 0.01), and interleukin-6 (IL-6) (*P* < 0.05), interleukin-8 (IL-8) (*P* < 0.05) and interleukin-10 (IL-10) (*P* < 0.01).

**Table 4:** The viral load, levels of inflammatory markers and lymphocyte subsets of the study subjects according to disease severity of COVID-19 during hospitalization

Laboratory findings	Disease severity of COVID-19			Statistic parameter	
(mean ± SD)	AS (n = 11)	MS (n = 26)	SCS (n = 11)	*F* value	*P* value
Ct values	29.9 ± 2.4	30.4 ± 1.9	31.5 ± 1.5	2.186	0.061
B cells (%)	15.0 ± 9.9	11.5 ± 7.0	4.0 ± 3.2	7.017	0.002**
NK cells (%)	9.8 ± 20.9	3.9 ± 2.9	3.4 ± 1.2	1.284	0.285
CD3 + T cells (%)	69.7 ± 9.9	62.7 ± 9.1	54.7 ± 13.9	5.640	0.007**
CD3 + CD4 + T cells (%)	37.5 ± 10.3	40.1 ± 7.9	31.6 ± 104	3.327	0.045*
CD3 + CD8 + T cells (%)	23.1 ± 6.3	24.2 ± 10.5	17.0 ± 5.6	5.315	0.008**
CD4 + /CD8 + ratio (%)	1.7 ± 1.3	2.3 ± 1.1	2.0 ± 0.9	1.219	0.305
IL2 (pg/mL)	5.4 ± 3.9	3.9 ± 3.6	6.7 ± 5.6	1.805	0.176
IL4 (pg/mL)	9.27 ± 20.9	3.9 ± 3.6	6.7 ± 5.6	1.335	0.273
IL5 (pg/mL)	6.7 ± 8.3	5.3 ± 5.6	5.8 ± 5.2	0.197	0.822
IL6 (pg/mL)	12.3 ± 18.4	6.1 ± 12.5	574.9 ± 1325.4	3.389	0.039*
IL8 (pg/mL)	132.5 ± 114.4	33.5 ± 43.7	869.7 ± 1927.2	3.374	0.043*
IL10 (pg/mL)	2.9 ± 1.1	2.7 ± 1.2	7.4 ± 7.5	6.525	0.003**
TNF-α (pg/mL)	30.9 ± 28.9	25.5 ± 38.0	15.2 ± 24.2	0.639	0.533
INF-α (pg/mL)	5.7 ± 9.3	4.7 ± 6.4	3.3 ± 0.8	0.380	0.686
INF-γ (pg/mL)	12.4 ± 24.0	19.2 ± 48.7	11.7 ± 8.7	0.211	0.810

One‐way analysis of variance. Statistically significant, *P < 0. 05, **P < 0.001

### Correlation between the age, viral loads, and levels of inflammatory markers and lymphocyte subsets

Table 5 demonstrates correlation between the age, viral loads, and levels of inflammatory markers and lymphocyte subsets on admission to hospital. The age was negatively correlated with CD 3 + T cells (*P* < 0.05), CD3 + CD4 + T cells (*P* < 0.05), and positively correlated with IL-2 (*P* < 0.001), IL-5 (*P* < 0.05), IL-6 (*P* < 0.05) IL-8 (*P* < 0.05), and IL-10 (*P* < 0.05). The viral loads were positively correlated with IL-2 (*P* < 0.001), IL-5 (*P* < 0.05), IL-6 (*P* < 0.05) IL-8 (*P* < 0.05) and IL-10 (*P* < 0.05), while negatively correlated with CD 3 + T cells (*P* < 0.05) and CD3 + CD4 + T cells (*P* < 0.05).

**Table 5:** Correlation between the age, viral loads, and levels of inflammatory markers and lymphocyte subsets in 48 cases

	n	Pearson’s correlation R	*P*-value
*Age*			
Viral loads	48	0.220	0.134
B cells (%)	48	− 0.145	0.327
NK cells (%)	48	0.077	0.605
CD 3 + T cells (%)	48	− 0.299	0.039*
CD3 + CD4 + T cells (%)	48	− 0.329	0.023*
CD3 + CD8 + T cells (%)	48	− 0.104	0.483
CD4 + /CD8 + ratio (%)	48	− 0.179	0.233
IL2 (pg/mL)	48	0.490	0.000**
IL4 (pg/mL)	48	0.175	0.233
IL5 (pg/mL)	48	0.325	0.024*
IL6 (pg/mL)	48	0.322	0.026*
IL8 (pg/mL)	48	0.286	0.047*
IL10 (pg/mL)	48	0.296	0.040*
TNF-α (pg/mL)	48	− 0.001	0.994
INF-α (pg/mL)	48	0.192	0.191
INF-γ (pg/mL)	48	0.183	0.213
*Viral loads*			
B cells (%)	48	0.070	0.637
NK cells (%)	48	0.074	0.615
CD 3 + T cells (%)	48	− 0.285	0.049*
CD3 + CD4 + T cells (%)	48	− 0.287	0.042*
CD3 + CD8 + T cells (%)	48	− 0.135	0.360
CD4 + /CD8 + ratio (%)	48	0.155	0.292
IL2 (pg/mL)	48	0.303	0.037*
IL4 (pg/mL)	48	0.338	0.019*
IL5 (pg/mL)	48	0.222	0.130
IL6 (pg/mL)	48	0.301	0.048*
IL8 (pg/mL)	48	0.298	0.049*
IL10 (pg/mL)	48	0.300	0.035*
TNF-α (pg/mL)	48	0.158	0.283
INF-α (pg/mL)	48	0.195	0.184
INF-γ (pg/mL)	48	− 0.027	0.856

**Correlation is significant at the 0.01 level (2-tailed). *P* < 0.001

*Correlation is significant at the 0.05 level (2-tailed). *P* < 0. 05

## Discussion

At present, preventing transmission of SARS-CoV-2 is very important. Asymptomatic SARS-CoV-2 patients may be the important source of infection [7, 9]. Asymptomatic infection is contagious, surveillance testing of asymptomatic persons is a critical strategy for the virus that causes COVID-19, and these patients may continue to test positive for the virus for up to 21 d. In the present study, 11 asymptomatic (AS), 26 mild (MS) and 11 severe or critical (SCS) SARS-CoV-2 infected patients were included. The mean age of AS group (47.3 years) was lower than SCS group (63.5 years) (*P* < 0.05). More than 60 years old in SCS group was 7 cases. Systemic and lung symptoms and chest CT changes in SCS group were more than MS group (*P* < 0.05). The age was negatively correlated with CD3 + T cells (*P* < 0.05), CD3 + CD4 + T cells (*P* < 0.05), and positively correlated with IL-2 (*P* < 0.001), IL-5 (*P* < 0.05), IL-6 (*P* < 0.05) IL-8 (*P* < 0.05), and IL-10 (*P* < 0.05). So the age may be one of causes of the severity of COVID-19. Because older patients (> 65 years old) mainly suffer from cardiovascular diseases, renal insufficiency and metabolic diseases (such as diabetes), and various organs of the whole body begin to age, so their resistance to pathogen invasion is poor, which makes their condition worse. Some drugs should have a certain effect on the COVID-9, but we only observed patients who were admitted to the hospital for 7 days. There were fewer samples, and the effect on SARS-CoV-2 may not be very obvious. In addition, certain non-communicable diseases such as diabetes and cardiovascular system diseases appear to increase the severity of COVID-19 and mortality risk, but SARS-CoV-2 infection in survivors with non-communicable diseases may also affect the progression of their pre-existing clinical conditions [11]. We have observed that the proportion of diabetes in patients with mild and severe cases with COVID-19 (MS and SCS group) was higher than that of AS group, and the concentration of IL6, IL8 and IL10 in MS and SCS groups was higher than that of AS group (*P* < 0.05). The basic and clinical science of the potential inter-relationships between diabetes mellitus and COVID-19 has been reviewed [11]. Initial studies found the presence of diabetes mellitus and the individual degree of hyperglycaemia seem to be independently associated with COVID-19 severity [11]. Nonetheless, the findings are consistent with some literature currently available regarding inflammatory biomarkers and clinical severity [12].

In the present study, viral loads of SARS-CoV-2 in AS were similar to MS and SCS on admission to hospital and during hospitalization. This is similar to the result of Ra et al. (2020) [7] and Chen et al. (2020) [9]. The SARS-CoV-2 is isolated from asymptomatic individuals and that a certain number of cases are likely to have been transmitted from asymptomatic individuals [6, 13, 14]. Since these AS may not be aware that they have been infected with SARS-CoV-2, and these individuals live in the community, and they have to go to work or school, or go shopping or exchange every day, community transmission and continued COVID-19 pandemic are inevitable. AS transmission should be one of the major challenges in controlling COVID-19 outbreak. Further studies on the epidemiological significance of these asymptomatic cases are warranted.

Exuberant inflammatory responses may promote T cell apoptosis, thus resulting in uncontrolled inflammatory responses due to diminished T cell responses. Our data also confirmed lower concentration of CD 3 + T cells (*P* < 0.05), CD3 + CD4 + T cells (*P* < 0.05), CD3 + CD8 + T cells (*P* < 0.01), and B cells (*P* < 0.05) in SCS patients than in AS and MS patients, while the level of IL-5 (*P* < 0.05), IL-6 (*P* < 0.05), IL-8 (*P* < 0.01) and IL-10 (*P* < 0.01), and TNF-α (*P* < 0.05) was higher on admission to hospital and during hospitalization. Additionally, the patient age and viral loads were seen to be significantly associated with lymphocyte subsets (negatively correlated) and inflammatory markers (positively correlated) by Pearson’s Correlation assay. No statistical differences were found in the level of IL4, INF-α, and INF-γ, and the percentage of NK cells, between three groups. This may be due to the small-sized sample in our study. In the present study, there were only 26 cases in MS group, while 11 cases were in the AS group and in the SCS group, respectively, on admission to hospital. Some parameter’s SD was lower than the mean. Therefore, we applied the ANOVA method for statistical analysis. Previous studies [2, 15] have shown that the SARS-CoV-2 infection may primarily affect T lymphocytes. The CD3 + , CD4 + and CD8 + T lymphocyte subsets were decreased in COVID-19-infected patients. When compared with the moderate patient and the severe patient, CD3 + , CD4 + and CD8 + T cells in the critical patient decreased greatly (Akbari et al., 2020; Liu et al., 2020) [2, 15]. Some studies have shown that elevated levels of pro-inflammatory cytokines, such as IFN-γ, TNF-a, IL-6 and IL-8, are associated with severe lung injury and adverse outcomes of SARS-CoV or MERS-CoV infection [16, 17]. In COVID**‐**19, the increased amounts of cytokines (e.g. IL‐1β, IFN‐γ, IP‐10, IL‐10 and IL‐4) is associated with COVID-19 severity [18]. Additionally, T cells are important for dampening overactive innate immune responses during viral infection. In our study, the viral loads were positively correlated with IL-2 (*P* < 0.001), IL-5 (*P* < 0.05), IL-6 (*P* < 0.05) IL-8 (*P* < 0.05) and IL-10 (*P* < 0.05), while negatively correlated with CD 3 + T cells (*P* < 0.05) and CD3 + CD4 + T cells (*P* < 0.05). Due to the rapid spread of COVID-19, asymptomatic leading to under-recognition of the related disease [19], and the high mortality rate of severely patients, it is necessary to better understand the clinical characteristics and identify reliable laboratory markers of inflammation in order to distinguish the mild, moderate and severe or critical infections.

### Limitations

Besides the low number of patients in our series, the main limitation of the current study is that in many laboratory inspections, we could not completely collect data such as levels of alanine aminotransferase, aspartate aminotransferase, creatine kinase MB activity, and lactate dehydrogenase which are associated with liver and myocardial injury, and we could not understand the patients' liver and heart function. In addition, although this was a retrospective study, because of large differences in enrollment time, some patients have not yet been discharged (only observed 5–7 days after admission), and their outcome and prognosis will still change (such as the patient improved or died).

## Conclusion

Among asymptomatic individuals (AS) and symptomatic patients (SCS) with COVID-19, no significant difference in viral loads was observed on admission to hospital and during hospitalization. The age and diabetes may be to increase the severity of COVID-19. The lymphocyte subset in AS were higher but cytokines were lower than SCS. It may be that asymptomatic patients are more resistant to the new coronavirus than symptomatic patients, and there is no obvious physical damage to themselves. But asymptomatic patients may be as an important source of infection for the COVID-19 and should be taken seriously.

## Data Availability

All data generated or analyzed during this study are included in this published article.

## Abbreviations

ANOVA: Analysis of variance
AS: Asymptomatic individuals
CDC: Centers for disease control and prevention
CD3 + T cells, CD4 + T cells and CD8 + T cells: Lymphocyte subset
COVID-19: Coronavirus disease-2019
CT: Computed tomography
Ct: Cycle threshold
IL-2: Interleukin-2
IL-4: Interleukin-4
IL5: Interleukin-5
IL-6: Interleukin-6
IL-8: Interleukin-8
IL-10: Interleukin-10
INF-γ: Interferon-gamma
MS: Mild symptoms
NK cells: Natural killer cells
RNA: Ribonucleic acid
RT-PCR: Reverse transcription polymerase chain reaction
SARS-CoV-2: Severe acute respiratory syndrome coronavirus 2
SCS: Symptomatic patients
SPSS: Statistical product and service solutions
TNF-α: Tumor necrosis factor-alpha
WHO: World Health Organization
